# Types of Entropies and Divergences with Their Applications

**DOI:** 10.3390/e25020198

**Published:** 2023-01-19

**Authors:** Nicuşor Minculete, Shigeru Furuichi

**Affiliations:** 1 Department of Mathematics and Computer Science, Transilvania University of Braşov, 500091 Braşov, Romania; 2 Department of Information Science, College of Humanities and Sciences, Nihon University, 3-25-40, Sakurajyousui, Setagaya-ku, Tokyo 156-8550, Japan

Entropy is an important concept in many fields related to communications. The concept of entropy was originally created by Shannon as part of his theory of communication, in which a data communication system is composed of three elements: a source of data, a communication channel, and a receiver. Many types of entropies and divergences have been studied in various works. The theory of entropy represents an old topic of many mathematical areas that still remain attractive research domains with many applications. The research results presented in this Special Issue concern the properties of different types of entropies and divergences, highlight their applications, and promote the exchange of ideas between mathematicians from many parts of the world. Entropies quantify the diversity, uncertainty, and randomness of a system. Many important types of entropies and divergences have applications in statistical mechanics, networks theory, quantum information theory, mathematical physics, mathematical analysis, etc. For example, the concept of Rényi entropy has been of great importance in statistics, ecology, theoretical computer science, etc.

Several manuscripts were selected for publication in this Special Issue that will be described in this article. These articles were prepared by scientists working in leading universities and research centers in Cuba, France, Germany, India, Japan, Mexico and Romania.

The α-divergence is related to the difference between the weighted arithmetic mean and the geometric mean. We mention that the gap is used in information geometry to define the Fenchel–Legendre divergence. S. Furuichi and N. Minculete, in their paper [1] “Refined Young Inequality and Its Application to Divergences”, established some bounds on the difference between the weighted arithmetic mean and the weighted geometric mean. These imply refined Young inequalities and the reverses of the Young inequality. The authors also studied some properties on the difference between the weighted arithmetic mean and the weighted geometric mean. Applying the newly obtained inequalities, the authors presented several results on the Tsallis divergence, the Rényi divergence, the Jeffreys–Tsallis divergence and the Jensen–Shannon–Tsallis divergence.

Entropy makes it possible to measure the uncertainty about an information source from the distribution of its output symbols. It is known that the maximum Shannon’s entropy of a discrete source of information is reached when its symbols follow a uniform distribution. In cryptography, these sources have great applications since they allow for the highest security standards to be reached. L. Contreras Rodrigues et al., in their paper [2] “Selecting an Effective Entropy Estimator for Short Sequences of Bits and Bytes with Maximum Entropy”, studied the most effective estimator to estimate entropy in short samples of bytes and bits with maximum entropy. For this, 18 estimators were compared. Results concerning the comparisons published in the literature between these estimators are discussed. The most suitable estimator is determined experimentally, based on its bias, from the mean square error short samples of bytes and bits.

The iterative probabilistic attack was proposed to reconstruct the internal state of the RC4 algorithm, starting from knowing an output sequence. This type of attack does not yet violate RC4, but it constitutes a serious potential threat to its security, which should not be ignored. Concerning this threat, a criterion has been developed to assess the vulnerability of an RC4 output to this type of attack. E.J. Madarro-Capó et al., in the paper [3] “Information Theory Based Evaluation of the RC4 Stream Cipher Outputs”, presented a criterion, based on information theory, to measure the amount of average information provided by the sequences of outputs of the RC4 on the internal state. The test statistic used is the sum of the maximum plausible estimates of the entropies $H(j_{t}|z_{t})$, corresponding to the probability distributions $P(j_{t}|z_{t})$ of the sequences of random variables ${\left(j_{t}\right)}_{t\in T}$ and ${\left(z_{t}\right)}_{t\in T}$, independent, but not identically distributed, where $z_{t}$ represents the known values of the outputs, while $j_{t}$ is one of the unknown elements of the internal state of the RC4. It is experimentally demonstrated that the test statistic allows for determining the most vulnerable RC4 outputs, and it is proposed to be used as a vulnerability metric for each RC4 output sequence concerning the iterative probabilistic attack.

A typical scenario for certifications on cryptographic algorithms such as AES is the estimation of attack success probability as a function of time or data availability. At the beginning of the last decade, a certification would usually include estimating the time necessary for recovering one secret byte from an AES implementation after running a side-channel attack. A. Tănăsescu et al., in the paper [4] “Tight and Scalable Side-Channel Attack Evaluations through Asymptotically Optimal Massey-like Inequalities on Guessing Entropy”, studied the bounds presented at CHES 2017 based on Massey’s guessing entropy, which represent the most scalable side-channel security evaluation method to date. In this paper, the authors presented an improvement on this method by determining the asymptotically optimal Massey-like inequality and then further refining it for finite support distributions. The impact of these results is highlighted for side-channel attack evaluations, demonstrating the improvements over the CHES 2017 bounds.

In [5], Massey studied a guessing problem where one is interested in the expected number of guesses required to guess a random variable *X* that assumes values from an infinite set and found a lower bound in terms of Shannon entropy. Arikan [6] studied it for a finite alphabet set and showed that Rényi entropy arises as the optimal solution in minimizing moments of the number of guesses. M. Ashok Kumar et al., in the paper [7] “Are Guessing, Source Coding and Tasks Partitioning Birds of A Feather?”, established a close relationship among the four information theoretic problems, namely Campbell source coding, Arikan guessing, Huleihel et al. memoryless guessing and Bunte–Lapidoth’s tasks partitioning problem in the i.i.d. lossless case. The authors first show that the aforementioned problems are mathematically related via a general moment minimization problem whose optimum solution is given in terms of Rényi entropy. They then propose a general framework for the mismatched version of these problems and establish all the asymptotic results using this framework. The unified framework further enables us to study a variant of Bunte–Lapidoth’s tasks partitioning problem, which is practically more appealing. In addition, this variant turns out to be a generalization of Arikan’s guessing problem. Finally, with the help of this general framework, they established an equivalence among all these problems, in the sense that knowing an asymptotically optimal solution in one problem helps us to find the same in all other problems.

This volume will be of interest to mathematicians specializing in information theory and beyond. Many of the results presented here may be very useful in demonstrating new results.
